# Janus Kinase Inhibitors for the Treatment of Atopic Dermatitis: Focus on Abrocitinib, Baricitinib, and Upadacitinib

**DOI:** 10.5826/dpc.1104a145

**Published:** 2021-10-01

**Authors:** Miguel Nogueira, Tiago Torres

**Affiliations:** 1 Department of Dermatology, Centro Hospitalar Universitário do Porto, Porto, Portugal; 2 Instituto de Ciências Biomédicas Abel Salazar, University of Porto, Porto, Portugal

**Keywords:** atopic dermatitis, treatment, janus kinase inhibitors, abrocitinib, baricitinib, upadacitinib

## Abstract

Atopic dermatitis (AD) is a clinically heterogenous, inflammatory skin condition with a high impact on patients’ daily activities that remains difficult to treat. The knowledge acquired over the last decade on AD pathophysiology and disease burden led to the development of new targeted therapeutic options that enable clinicians to better manage AD patients. The JAK/STAT signaling pathway modulates several immune pathways (T helper (Th)1, Th2, Th17, and Th22 cells) that have been found to be involved in AD pathogenesis. For this reason, JAK inhibitors emerged as a possible therapy for AD. Baricitinib, upadacitinib, and abrocitinib are the three oral JAK inhibitors already approved or in advanced clinical development for this purpose. The results showed that this drug class is highly effective achieving symptomatic relief (itch control) in the short term, as well as improving disease severity in the short and medium term. However, their efficacy should be balanced with possible side effects, that have been reported in clinical trials. More data on the long-term efficacy and safety, as well as from head-to-head comparisons and from real-world setting will be crucial to position oral JAK inhibitors in the AD therapeutic armamentarium.

## Introduction

Atopic Dermatitis (AD) is a chronic, relapsing, clinically heterogeneous, and difficult-to-treat disorder, also known for being the most prevalent inflammatory skin disease in developed countries with a lifetime prevalence of up to 20% [1,2]. AD was originally labeled as an early childhood disease. However, it is now known that despite the incidence peak during infancy – onset of the disease occurs before 6 years of age in 80% of patients -, the proportion of patients with adult-persistent or adult-onset forms of AD is nonnegligible [2–4].

The disease is clinically associated with a variety of features ranging from minimal eczema on flexural folds or hands to erythroderma [2–4]. Cutaneous manifestations and associated symptoms/signals, such as redness, peeling, lichenification, and itchiness markedly interfere with daily activities and may lead to sleep loss and diminished self-esteem, having a high impact on social interactions and school/work performance [2–4]. Consequently, AD is an important health concern worldwide, occupying the top position on the list of the causes of non-fatal disease burden of skin disorders [3].

The pathophysiology of AD is thought to be a result of the interaction between various critical factors, such as epidermal barrier disruption, genetic susceptibility, activation of distinct subsets of T-cells, environmental triggers, and dysbiosis of commensal skin microbiota [2,5]. The strongest identified risk factor for AD is a positive family history of atopy which may result in a 3 or 5-fold increased risk for developing AD when one or both parents are affected, respectively [2].

In the last decade, major advances were made in understanding the molecular bases behind AD, which also allowed the development of new targeted strategies for the management of the disease [2,6,7]. In addition to the classic treatment approaches – including avoiding environmental triggers, topical therapies and traditional immunosuppressants – several targeted drugs (biologic or small molecules) were introduced as new treatment options for AD. Dupilumab, an IL-4 receptor α inhibitor that blocks the activity of both IL-4 and IL-13, was the first non-traditional immunomodulator agent approved for the treatment of moderate-to-severe AD in patients with 6 years of age or older. However, the development of new treatments for AD is crucial, as a considerable number of patients are non-responders to the available regimens. Currently, several small molecules are already approved or under evaluation in clinical trials for treating patients with AD [4,6–9].

### Janus Kinase (JAK)/Signal Transducer and Activator of Transcription (STAT) Signaling Pathway in Atopic Dermatitis

The JAK/STAT signaling pathway has been associated with the development of AD as it modulates several immune pathways that are involved in its pathogenesis, such as those associated with T helper (Th)1, Th2, Th17, and Th22 cells [10, 11]. Briefly, this pathway involves a circulating cytokine, such as an interferon (IFN) or an interleukin (IL), that binds to its cell membrane receptor triggering a conformational change with subsequent JAKs recruitment and activation [12]. Two activated and combined JAKs have the ability to further phosphorylate STAT proteins, inducing their dimerization and subsequent translocation into the cell nucleus. Once in the nucleus, STAT proteins can affect gene expression [12]. There are four different JAK proteins (JAK1, JAK2, JAK3, and TYK2) and seven distinct STAT proteins (STAT1, STAT2, STAT3, STAT4, STAT5a, STAT5b, and STAT6) [12]. JAK1 and JAK3 seem to be responsible for mediating the signaling of cytokines from the γc family, including IL-4, a cytokine known for its relevant role in AD cascade [10,11]. IL-31-induced signaling pathway, particularly through JAK1, is also thought to play an important role in inducing chronic pruritus [11,13]. STAT6 activation, which occurs mainly through JAK1 and JAK3 activation after IL-4 and IL-13 bind their receptors, have also been implicated in AD development [10,11,14,15]. On the other hand, JAK2 and TYK2 interact to STAT4 and seem to mediate Th1 differentiation, as well as IL-12 signaling [10,11].

### Systemic JAK inhibitors in Atopic Dermatitis

Over the last decade, the marked advances that have been made in understanding the impact of JAK/STAT signaling pathway on the pathogenesis of AD encouraged the use of JAK inhibitors as a therapeutic alternative for this disease [10,11,16]. Although JAK inhibitors safety profile had already been evaluated - this class of drugs is already approved for the treatment of other immune mediated diseases such as rheumatoid arthritis – several clinical trials were conducted to assess the performance of these drugs in the specific context of AD[10,11].

In the following review, we will focus on the results of the available phase III clinical trials of oral JAK inhibitors used in AD patients after their positive data on safety and efficacy in phase I and II studies, namely baricitinib, upadacitinib and abrocitinib (Table 1).

#### Baricitinib

Baricitinib is a small molecule that selectively inhibits both JAK1 and JAK2 proteins [10,11]. This drug is approved by the European Medicines Agency (EMA) for treating moderate-to-severe atopic dermatitis in adult patients, but is currently waiting for the approval of Food and Drugs Administration (FDA) [17]. The recommended dosage is 4 mg once daily. Half dosage schemes (2 mg) may be considered in patients aged 75 years or older, in those with a history of chronic or recurrent infections, or in those who have achieved a sustained control of disease as a dose tapering strategy [17]. As these are the recommended dosage regimens, data on the usage of 1 mg dosage schemes will not be discussed.

Two independent multicentric double-blinded 16-week phase III clinical trials (BREEZE-AD1 and BREEZE-AD2) compared baricitinib monotherapy (2 mg and 4 mg) with placebo in adult patients with moderate-to-severe AD [18]. The results of both trials revealed that the primary endpoint [Validated Investigator’s Global Assessment for AD (vIGA-AD) score of 0 (clear) or 1 (almost clear)] was achieved with both baricitinib 2 mg and 4 mg once daily dosing-regimens [BREEZE-AD1: N = 624, baricitinib 2 mg 11.4 %, baricitinib 4 mg 16.8 % vs. placebo 4.8 % (P < 0.05 and P < 0.001, respectively); BREEZE-AD2: N = 615, baricitinib 2 mg 10.6 %, baricitinib 4 mg 13.8 % vs. placebo 4.5 % (P < 0.05 and P = 0.001, respectively)]. However, the same was not verified for secondary endpoints, as only baricitinib 4 mg consistently met the key secondary endpoints. Although improvements in AD-associated symptoms (skin pain and night awakenings) were achieved at week 1 for both dosing regimens, the same was not verified for itchiness - it improved at week 1 with baricitinib 4 mg, but patients receiving baricitinib 2 mg only registered improvements at week 2, making the first dosing regimen superior. Treatment-emergent adverse events were observed in 54 to 58 % of the patients, predominantly mild-to-moderate. The most reported adverse events included increased blood creatine phosphokinase (CPK), nasopharyngitis and headache. Herpes Simplex infection was more frequent with baricitinib compared to placebo in BREEZE-AD1 (baricitinib 2 mg 3.3 %, baricitinib 4 mg 7.2 % vs. placebo 1.2 %), but the same was not verified in BREEZE-AD2. No venous thromboembolic events, significant hematological abnormalities, cardiovascular events, or death were observed in any of the baricitinib groups.

After a 16-week period, adults receiving baricitinib 4 mg or 2 mg that were responders or partial responders (vIGA-AD score ≤ 2) in BREEZE-AD1 and BREEZE-AD2 trials were analyzed in a long-term extension study (BREEZE-AD3) [19]. The proportion of patients receiving baricitinib 2 mg and 4 mg that achieved a vIGA-AD of 0 or 1 at week 16 (BREEZE-AD3 study baseline) was 46.3 % and 45.7 %, respectively. At week 68, those numbers improved to 59.3 % and 47.1 %, with a similar safety profile, thus showing the sustained long-term efficacy and safety of baricitinib in these patients.

The combination of baricitinib (2 mg and 4 mg) with topical corticosteroids was evaluated in two distinctive phase III clinical trials (BREEZE-AD4 [20] and BREEZE-AD7 [21]) that included adult patients with moderate-to-severe AD. In these trials, only baricitinib 4 mg met the primary endpoint [an improvement of 75 % in Eczema Area and Severity Index (EASI) compared to baseline (EASI75) in BREEZE-AD4, and a vIGA-AD score of 0/1 in BREEZE-AD7] at week 16. At the mentioned timepoint, a total of 31.5 % of patients receiving baricitinib 4 mg achieved EASI75 (baricitinib 2 mg 27.6 %; placebo 17.2 %) in BREEZE-AD4, while 31.0 % achieved vIGA-AD score of 0/1 (baricitinib 2 mg 24.0 %; placebo 15.0 %) in BREEZE-AD7. The symptomatic relief (improvement in itch scores) was verified in all patients receiving baricitinib – when comparing to placebo, a higher response of itch improvement with baricitinib 4 mg was achieved as early as on day 4 of treatment. Regarding side effects, infections and increased blood CPK were the most common reported ones. A pulmonary thromboembolic event in a patient receiving baricitinib 4 mg was reported in BREEZE-AD7. The impact of the combined therapy in the health-related quality of life and productivity of AD patients, as well as the improvement in patient-reported outcomes were also assessed in BREEZE-AD7 [22]. Both dosing regimens (2 mg and 4 mg) of baricitinib plus topical corticosteroids induced a rapid – before week 2 – and significant improvement in several of the evaluated scores (Dermatology Life Quality Index, Work Productivity and Activity Impairment, Patient-Reported Outcomes Measurement Information System Itch and Sleep) when compared to placebo plus topical corticosteroids in the same timepoints.

#### Upadacitinib

Upadacitinib is a small molecule that selectively inhibits JAK1 [23]. The drug is approved by the EMA for the treatment of moderate-to-severe AD in patients aged 12 years or older [23]. Similarly to baricitinib, upadacitinib is currently under FDA evaluation. The recommended dose is 15 mg or 30 mg once daily, according to the disease burden and age of the patient [23].

Two 16-week phase III clinical trials (Measure Up 1 and Measure Up 2) compared upadacitinib monotherapy with placebo in patients aged 12 to 75 years with moderate-to-severe AD [24]. A total of 847 patients in Measure Up 1 and 836 patients in Measure Up 2 were randomly (1:1:1 ratio) assigned to receive either upadacitinib 15 mg, upadacitinib 30 mg, or placebo. The coprimary endpoints at week 16 (EASI75 and vIGA-AD score of 0/1) were achieved in all upadacitinib groups in both Measure Up 1 [EASI75: upadacitinib 15 mg 69.6 %, upadacitinib 30 mg 79.9 % vs. placebo 16.3 % (P < 0.0001); vIGA-AD score of 0/1: upadacitinib 15 mg 48.1 %, upadacitinib 30 mg 62.0 % vs. placebo 8.4 % (P < 0.0001)] and Measure Up 2 [EASI75: upadacitinib 15 mg 60.1 %, upadacitinib 30 mg 72.9 % vs. placebo 13.3 % (P < 0.0001); vIGA-AD score of 0/1: upadacitinib 15 mg 38.8 %, upadacitinib 30 mg 52.0 % vs. placebo 4.7 % (P < 0.0001)]. The secondary endpoints at week 16 were also met in both upadacitinib groups. There was a similar incidence of serious adverse events and adverse events leading to study dropout between groups. Acne, upper respiratory tract infections, nasopharyngitis, headache, and increase in serum CPK levels were the most frequently reported adverse events. Regarding herpes zoster, the rates of infection in the groups receiving upadacitinib were low (< 2 %).

A 52-week phase III trial (AD Up) evaluated the efficacy and safety of combining upadacitinib with topical corticosteroids in patients aged 12 to 75 years old [25,26]. In a 1:1:1 ratio randomization, 901 patients were assigned to receive topical corticosteroids plus either upadacitinib 15 mg, upadacitinib 30 mg or placebo [25,26]. The coprimary endpoints at week 16 (EASI75 and vIGA-AD score of 0/1) were achieved in the two upadacitinib groups [EASI75: upadacitinib 15 mg 64.6 %, upadacitinib 30 mg 77.1 % vs. placebo 26.4 % (P < 0.0001); vIGA-AD score of 0/1: upadacitinib 15 mg 39.6 %, upadacitinib 30 mg 58.6 % vs. placebo 10.9 % (P < 0.0001)], and both upadacitinib groups achieved higher response rates than placebo for all key secondary endpoints [25]. Not only upadacitinib (both doses) demonstrated to have a higher efficacy, but also a rapid onset of action, with differences observed as early as week 2 [25]. Although similar rates of herpes zoster infections were observed across groups (1 to 2 %), eczema herpeticum was only registered in patients receiving upadacitinib [25]. No new safety findings were reported at week 16 [25]. For all endpoints, efficacy of both dosing regimens of upadacitinib were maintained through week 52 [26]. No new relevant safety events were reported in the extended period [26].

Upadacitinib was also tested in a 24-week head-to-head phase III clinical trial (Heads Up) [27]. A total of 692 adult patients with moderate-to-severe AD were randomized (1:1 ratio) to receive either upadacitinib 30 mg once daily or dupilumab 300 mg every other week (600 mg as initial loading dose). At week 16, 71.0 % patients in the upadacitinib group achieved EASI75, compared to 61.1 % in the dupilumab one (P = 0.006). In addition, upadacitinib demonstrated to have a faster onset of action when compared to dupilumab: 43.7 % of patients receiving upadacitinib achieved EASI75 at week 2, compared to 17.4 % of those receiving dupilumab. No new safety-related events were registered compared to the already available data for both drugs. It was verified a higher rate of serious infections, herpes zoster, eczema herpeticum, and laboratory adverse events in the group of patients receiving upadacitinib whereas rates of conjunctivitis and injection-site reactions were higher in the dupilumab group. One treatment-emergent death occurred in the upadacitinib group due to a viral bronchopneumonia.

#### Abrocitinib

Abrocitinib is a JAK1 selective inhibitor that, differently from the previous drugs, has neither received FDA or EMA approval yet. FDA is now reviewing the New Drug Application for abrocitinib, and a decision is expected soon after.

Two 12-week similarly designed phase III trials (JADE MONO-1 and JADE MONO-2) [28,29] compared abrocitinib monotherapy (100 mg and 200 mg once daily) with placebo in a total of 778 patients with moderate-to-severe AD aged 12 years or older. Both dosing regimens of abrocitinib met the coprimary endpoints at week 12 (EASI75 and Investigator Global Assessment (IGA) score of 0 or 1) in both JADE MONO-1 [EASI75: abrocitinib 100 mg 40 %, abrocitinib 200 mg 63 % vs. placebo 12 % (P < 0.0001); IGA score of 0/1: abrocitinib 100 mg 24 %, abrocitinib 200 mg 44 % vs. placebo 8 % (P = 0.0037 and P <0.0001, respectively)] and JADE MONO-2 [EASI 75: abrocitinib 100 mg 45 %, abrocitinib 200 mg 61 % vs. placebo 10 % (p < 0.001); IGA score of 0/1: abrocitinib 100 mg 28 %, abrocitinib 200 mg 38 % vs. placebo 9 % (p < 0.001)]. Regarding safety, treatment-emergent adverse events were more frequently registered in patients receiving abrocitinib in both trials (69 – 78 % in abrocitinib groups and 57 % in placebo group in JADE MONO-1; 63 – 66 % in abrocitinib groups and 42 % in placebo group in JADE-MONO-2), but there was a similar rate of serious adverse events with abrocitinib when comparing to the placebo group. Despite the low rates, herpes simplex infection was only observed in patients receiving abrocitinib. Nausea, nasopharyngitis, and headache were the most commonly reported adverse events, but there was also a relevant mention to dose-dependent acne after abrocitinib. No deaths occurred. Patients that completed 16 weeks of treatment in JADE MONO-1 and JADE MONO-2 were invited to enroll an ongoing phase III long-term extension study (JADE EXTEND – NCT03422822) including 92 weeks of treatment with abrocitinib (100 mg or 200 mg) with or without concomitant topical corticosteroids.

An active-controlled 16-week phase III clinical trial (JADE COMPARE) enrolled a total of 838 adult patients with moderate-to-severe AD and compared the efficacy and safety of abrocitinib (100 mg or 200 mg) with dupilumab 300 mg every other week (600 mg of initial loading dose) and placebo [30]. In this study, all patients received concomitant topical corticosteroids. EASI75 was achieved by 58.7 % of patients in abrocitinib 100 mg group, 70.3 % in abrocitinib 200 mg group, 58.1 % in dupilumab group, and 27.1 % in placebo group. The goal of an IGA score of 0/1 was achieved by 36.6 % in the abrocitinib 100 mg group, 48.4 % in the abrocitinib 200 mg group, 36.5 % in the dupilumab one, and 14.0 % in the placebo group (P < 0.001 for both abrocitinib groups compared to placebo). Abrocitinib 200 mg (but not abrocitinib 100 mg) was superior to dupilumab in achieving the itch-relief-scores at week 2. However, at week 16, none of abrocitinib groups differed significantly from the dupilumab one regarding other key secondary endpoints. In what concerns to safety questions, herpes zoster only occurred in patients receiving abrocitinib, while eczema herpeticum developed in 2 patients, one from the abrocitinib 100 mg group, and the other from the placebo group. No new safety-related events were noted. Patients that completed this trial were also invited to enroll the JADE EXTEND (NCT03422822).

In a 52-week phase III clinical trial (JADE REGIMEN), patients with moderate-to-severe AD aged 12 years or older that responded positively (achieved EASI75 and IGA score of 0/1) to a 12-week induction period of an open-label treatment with abrocitinib 200 mg once daily, were randomly assigned to receive blinded abrocitinib (100 mg or 200 mg) or placebo for 40 weeks [31]. A total of 1233 patients started at week 0, 798 (64.7 %) responded to the 12-week induction period and were randomly assigned to the abovementioned groups. The primary endpoint – proportion of patients that did not loss EASI50 and had IGA score < 3 (mild) – was achieved by 57.4 % in abrocitinib 100 mg group, 81.1 % patients in abrocitinib 200 mg group, and 19.1 % in placebo group, with statistically superiority of abrocitinib 200 mg compared to abrocitinib 100 mg. Regarding safety, treatment-emergent adverse events (abrocitinib 100 mg 54.0 %, abrocitinib 200 mg 63.2 %) and adverse events leading to drug discontinuation (abrocitinib 100 mg 1.9 %, abrocitinib 200 mg 6.0 %) were higher with abrocitinib.

A total of 285 adolescents aged 12 to 17 years with moderate-to-severe AD were enrolled in a 12-week phase III placebo-controlled study (JADE TEEN) and randomized to receive either once daily abrocitinib 100 mg, abrocitinib 200 mg, or placebo, in combination with topical corticosteroids [32]. The coprimary endpoints (EASI75 and IGA score of 0/1) were achieved in both abrocitinib groups [EASI75: abrocitinib 100 mg 68.5 %, abrocitinib 200 mg 72.0 % vs. placebo 41.5 % (P < 0.05); IGA score of 0/1: abrocitinib 100 mg 41.6 %, abrocitinib 200 mg 46.2 % vs. placebo 24.5 % (P < 0.05)]. Adverse events were observed in 52.0 % to 63.0 % of the patients among all groups, and the most reported were nausea, upper respiratory tract infections, headache, and nasopharyngitis. Herpes simplex infection was uncommon (1.1 %). No new safety-related events were noted when compared to the available data from previous studies.

## Conclusion

The results of the aforementioned trials revealed that JAK inhibitors are an effective therapeutic option for AD. Not only they were able to induce short and medium-term cutaneous improvement, but also to induce symptomatic relief, confirmed by their impact on itch control and on patients’ daily activities previously affected by AD. Another important-to-mention finding was the rapid onset of action of JAK inhibitors, able of inducing their benefits after just 1 to 2 weeks of treatment. These findings support the fact that an improvement in AD patients goes beyond the sole cutaneous improvement. Additionally, available data on head-to-head comparisons suggested that JAK inhibitors might be an important option in AD armamentarium, with a slightly numerical superiority in efficacy when compared to the already FDA and EMA approved dupilumab.

However, their potential side effects must be considered when opting for these drugs. In fact, JAK/STAT signaling pathway has been shown to play a role in several crucial physiological regulatory processes, that potentially increases their risk of side effects. Although most of them are mild-to-moderate, they include infections – including infection by opportunistic agents such as Mycobacterium tuberculosis or Herpes Zoster –, thromboembolic events, malignancies, lipid disturbances (increased total, low- and high-density lipoprotein cholesterol and increased triglycerides), and hematologic abnormalities (neutropenia, anemia, thrombocytopenia), and transient increases of liver enzymes, creatine phosphokinase (CPK), or creatinine [10,11]. Although hematologic disturbances have also been reported after the use of JAK1 inhibitors – by a mechanism that is not completely understood -, they are more commonly observed after using JAK2 inhibitors as JAK2 interferes with erythropoietin and other colony-stimulating factors (CSF) function [10,11]. Regarding lipid abnormalities, they have been mostly reported after the use of JAK1 inhibitors, however more data is needed to better clarify this interaction as some reports suggest that lipid disturbances may be related to inflammation and not to JAK1 pathway itself [10,11].

Over the last decade, knowledge on AD pathophysiology and its impact in quality of life has expanded, which promoted the pursuit for new targeted therapeutic options that enable clinicians to better manage their AD patients. Dupilumab was the first biological agent approved for moderate-to-severe AD, and data from clinical trials and real-world setting studies have been extensively analyzed and discussed in the recent years [33]. However, targeted therapy continued to evolve, and the emergence of small molecules, such as the JAK inhibitors baricitinib, upadacitinib, and abrocitinib, is increasing the range of options for dermatologists to treat their patients. JAK inhibitors seem to have a faster onset of action than alternative agents, such as dupilumab, although the latter have a more favorable safety profile with no requirements of blood monitoring. Despite the efficacy and safety profile that JAK inhibitors demonstrated in clinical trials, some questions remain, including their long-term efficacy (rate of flares), long-term safety events, head-to-head comparisons, and data on the efficacy and safety of the drug in a real-world setting.

In summary, the improvement on the knowledge of the pathophysiology of AD is allowing the targeting of the treatment of patients on a molecular basis [11,34–37]. New emergent drugs are allowing a smoother balance between efficacy and side effects [11,34–37]. These new drugs are crucial to improve success in AD management. This applies particularly to non-responder patients, that represent an important group whose needs have to be addressed.

## Figures and Tables

**Table 1:** Results From Phase III Clinical Trials of Oral JAK Inhibitors Used for AD Patients: Baricitinib, Upadacitinib and Abrocitinib.

Drug name	Main JAK inhibition	Trial Name	Patients, n.	Study duration	(Co)Primary Endpoint(s)	Most important side effects associated to JAK inhibitors
**Baricitinib**	*JAK1 and JAK2*	*BREEZE-AD1 [18]*	624	16 wks	*vIGA-AD 0/1 at week 16* - baricitinib 2mg: 11.4% - baricitinib 4mg: 16.8% - placebo: 4.8%	Increased blood CPK, nasopharyngitis, headache, herpes simplex
		*BREEZE-AD2 [18]*	615	16 wks	*vIGA-AD 0/1 at week 16* - baricitinib 2mg: 10.6% - baricitinib 4mg: 13.8% - placebo: 4.5%	Increased blood CPK, nasopharyngitis, headache
		*BREEZE-AD3 [19]*	221 total/partial responders	(16 wks from previous enrollment in BREEZE-AD1 or BREEZE-AD2) plus 52 wks	*vIGA-AD 0/1 at week 16, 36 and 52* Published results at week 52: - baricitinib 2mg: 59.3% - baricitinib 4mg: 47.1%	Nasopharyngitis, headache, increased blood CPK and diarrhea
		*BREEZE-AD4 [20]*	463	16 wks	*EASI75 at week 16* - baricitinib 2mg+TCS: 27.6% - baricitinib 4mg+TCS: 31.5% - placebo+TCS: 17.2%	Nasopharyngitis, headache, upper tract respiratory infections
		*BREEZE-AD7 [21]*	329	16 wks	*vIGA-AD 0/1 at week 16* - baricitinib 2mg+TCS: 24.0% - baricitinib 4mg+TCS: 31.0% - placebo+TCS: 15.0%	Nasopharyngitis, folliculitis, Herpes Simplex infection, upper respiratory tract infection, acne, diarrhea, and back pain
**Upadacitinib**	*JAK1*	*Measure Up 1 [24]*	847	16 wks	*EASI75 at week 16* - upadacitinib 15mg: 69.6% - upadacitinib 30mg: 79.9% - placebo: 16.3% *vIGA-AD 0/1 at week 16* - upadacitinib 15mg: 48.1% - upadacitinib 30mg: 62.0% - placebo: 8.4%	Acne, upper respiratory tract infections, nasopharyngitis, headache and increased blood CPK
		*Measure Up 2 [24]*	836	16 wks	*EASI75 at week 16* - upadacitinib 15mg: 60.1% - upadacitinib 30mg: 72.9% - placebo: 13.3% *vIGA-AD 0/1 at week 16* - upadacitinib 15mg: 38.8% - upadacitinib 30mg: 52.0% - placebo: 4.7%	Acne, upper respiratory tract infections, nasopharyngitis, headache and increased blood CPK
		*AD Up [25,26]*	901	52 wks	*EASI75 at week 16* - upadacitinib 15mg+TCS: 64.6% - upadacitinib 30mg+TCS: 77.1% - placebo+TCS: 26.4% *vIGA-AD 0/1 at week 16* - upadacitinib 15mg+TCS: 39.6% - upadacitinib 30mg+TCS: 58.6% - placebo+TCS: 10.9%	Acne, nasopharyngitis, increased blood CPK, upper respiratory tract infection, Herpes Simplex infection
		*Heads Up [27]*	692	24 wks	*EASI75 at week 16* - upadacitinib 30mg: 71.0% - dupilumab: 61.1%	Acne, upper respiratory tract infections, nasopharyngitis, increased blood CPK
**Abrocitinib**	*JAK1*	*JADE MONO-1 [28]*	387	12 wks	*EASI75 at week 12* - abrocitinib 100mg: 40.0% - abrocitinib 200mg: 63.0% - placebo: 12.0% *IGA 0/1 at week 12* - abrocitinib 100mg: 24.0% - abrocitinib 200mg: 44.0% - placebo: 8.0%	Nausea, nasopharyngitis, headache, upper respiratory tract infection
		*JADE MONO-2 [29]*	391	12 wks	*EASI75 at week 12* - abrocitinib 100mg: 45.0% - abrocitinib 200mg: 61.0% - placebo: 10.0% *IGA 0/1 at week 12* - abrocitinib 100mg: 28.0% - abrocitinib 200mg: 38.0% - placebo: 9.0%	Nausea, nasopharyngitis
		*JADE COMPARE [30]*	838	16 wks	*EASI75 at week 12 (vs. placebo)* - abrocitinib 100mg+TCS: 58.7% - abrocitinib 200mg+TCS: 70.3% - dupilumab: 58.1% - placebo+TCS: 27.1% *IGA 0/1 at week 12 (vs. placebo)* - abrocitinib 100mg+TCS: 36.6% - abrocitinib 200mg+TCS: 48.4% - dupilumab: 36.5% - placebo+TCS: 14.0%	Nausea, nasopharyngitis, upper respiratory tract infection, headache, acne, Herpes Zoster, thrombocytopenia
		*JADE REGIMEN [31]*	1233 (798 after a 12-week induction period with abrocitinib 200mg)	52 wks	*Proportion of patients that did not loss EASI50 and had IGA score <3 at week 52* - abrocitinib 100mg: 57.4% - abrocitinib 200mg: 81.1% - placebo: 19.1%	Nausea, nasopharyngitis, acne, upper respiratory tract infections, increased blood CPK
		*JADE TEEN [32]*	285	12 wks	*EASI75 at week 12* - abrocitinib 100mg+TCS: 68.5% - abrocitinib 200mg+TCS: 72.0% - placebo+TCS: 41.5% *IGA 0/1 at week 12* - abrocitinib 100mg+TCS: 41.6% - abrocitinib 200mg+TCS: 46.2% - placebo+TCS: 24.5%	Nausea, upper respiratory tract infections, headache, and nasopharyngitis

CPK = creatine phosphokinase; EASI = Eczema Area and Severity Index; EASI-50 = Improvement of 50% in EASI compared to baseline; EASI-75 = Improvement of 75% in EASI compared to baseline; IGA 0/1 = Investigator Global Assessment score of 0 or 1; JAK = Janus Kinase; TCS = topical corticosteroids; vIGA-AD 0/1 = Validated Investigator’s Global Assessment for Atopic Dermatitis score of 0 or 1; wks = weeks
